# Residual volume/total lung capacity ratio confers limited additive significance to lung clearance index for assessment of adults with bronchiectasis

**DOI:** 10.1371/journal.pone.0183779

**Published:** 2017-09-08

**Authors:** Wei-jie Guan, Jing-jing Yuan, Yan Huang, Hui-min Li, Rong-chang Chen, Nan-shan Zhong

**Affiliations:** 1 State Key Laboratory of Respiratory Disease, National Clinical Research Center for Respiratory Disease, Guangzhou Institute of Respiratory Disease, First Affiliated Hospital of Guangzhou Medical University, Guangzhou, Guangdong, China; 2 Sino-French Hoffmann Institute, Guangzhou Medical University, Guangzhou, China; University Children`s Hospital Zurich, SWITZERLAND

## Abstract

**Background:**

Mosaicism and hyperinflation are common pathophysiologic features of bronchiectasis. The magnitude of ventilation heterogeneity might have been affected by the degree of hyperinflation. Some studies have evaluated the discriminative performance of lung clearance index (LCI) in bronchiectasis patients, but the additive diagnostic value of hyperinflation metrics to LCI is unknown.

**Objective:**

To compare LCI and the ratio of residual volume to total lung capacity (RV/TLC), along with the LCI normalized with RV/TLC, in terms of discriminative performance, correlation and concordance with clinical variables in adults with bronchiectasis.

**Methods:**

Measurement items included chest high-resolution computed tomography, multiple-breath nitrogen washout test, spirometry, and sputum culture. We analyzed bronchodilator responses by stratifying LCI and RV/TLC according to their median levels (LCI^High^/RV/TLC^High^, LCI^Low^/RV/TLC^High^, LCI^High^/RV/TLC^Low^, and LCI^Low^/RV/TLC^Low^).

**Results:**

Data from 127 adults with clinically stable bronchiectasis were analyzed. LCI had greater diagnostic value than RV/TLC in discriminating moderate-to-severe from mild bronchiectasis, had greater concordance in reflecting clinical characteristics (including the number of bronchiectatic lobes, radiological severity score, and the presence of cystic bronchiectasis). Normalization of LCI with RV/TLC did not contribute to greater discriminative performance or concordance with clinical variables. The LCI, before and after normalization with RV/TLC, correlated statistically with age, sex, HRCT score, *Pseudomonas aeruginosa* colonization, cystic bronchiectasis, and ventilation heterogeneity (all P<0.05). Different bronchodilator responses were not significant among the four subgroups of bronchiectasis patients, including those with discordant LCI and RV/TLC levels.

**Conclusion:**

LCI is superior to RV/TLC for bronchiectasis assessment. Normalization with RV/TLC is not required. Stratification of LCI and RV/TLC is not associated with significantly different bronchodilator responses.

## Introduction

Ventilation heterogeneity, which exists in physiologic conditions [1], aggravates in chronic airway diseases including cystic fibrosis (CF) [2] and bronchiectasis [3–6]. The underlying causes for worsening ventilation heterogeneity are multifaceted, including mucus plugging [5] and airway remodeling [7] which are cascades of lung infections [8,9]. Such events can be readily observed in bronchiectasis regardless of the etiology (e.g. post-tuberculous infection) [3]. Lung clearance index (LCI) has been shown to sensitively detect ventilation heterogeneity and correlate with disease severity metrics, such as high-resolution computed tomography (HRCT) scores, in bronchiectasis [3–6]. Compared with spirometry, LCI conferred greater sensitivity in identifying early-stage lung structural disorders, which are likely related to small airways [3]. LCI has also been shown to be a sensitive, repeatable and practical parameter for assessing airway disorders in adults with cystic fibrosis [10].

Hyperinflation correlates with airway remodeling and disease severity in COPD [11,12]. In patients with bronchiectasis, airway destruction and remodeling may have predisposed to lung hyperinflation, which could be partially reflected by the functional residual capacity, measured with multiple-breath gas washout tests (MBW) [13]. The ratio of residual volume to total lung capacity (RV/TLC) is a more comprehensive parameter that could be readily calculated based on the functional residual capacity. Compared with HRCT-related hyperinflation metrics, RV/TLC can be inferred from the functional residual capacity measured with MBW, which measures the LCI with no potential risk of radiation exposures. It is hypothesized that more severe hyperinflation might contribute to greater magnitude of ventilation heterogeneity because of greater dead space volume which hinders regional ventilation and/or gas exchange. Currently, it remains unclear whether inhaled bronchodilators (such as salbutamol) result in a different magnitude of improvement in hyperinflation and ventilation heterogeneity. How the two metrics correlate with, and differ from, each other in bronchiectasis has not been documented.

Our research hypothesis was that LCI correlated with the RV/TLC, that LCI might be affected the RV/TLC, and that adjustment with RV/TLC could potentially improve the diagnostic performance of LCI in patients with bronchiectasis. Therefore, we investigated the discriminative performance of RV/TLC and LCI, and the concordance and correlation with clinical parameters. We normalized LCI with RV/TLC to determine whether this would improve the discriminative performance of LCI. Finally, we assessed bronchodilator responses by stratifying the RV/TLC and LCI according to their median values (LCI^High^/RV/TLC^High^, LCI^Low^/RV/TLC^High^, LCI^High^/RV/TLC^Low^, and LCI^Low^/RV/TLC^Low^). These might help address the question whether RV/TLC should be taken into account when LCI was adopted for assessment of ventilatory heterogeneity in patients with bronchiectasis.

## Methods

### Patients

Enrollment of consecutive bronchiectasis patients was conducted between March 2014 and May 2016. Diagnosis of bronchiectasis was made according to chest HRCT, effective within 12 months. Patients were aged 18 years or greater, and had no exacerbation for more than 4 weeks. Exacerbation should meet three or more criteria (persisting for >24hrs): significantly increased cough frequency; increased sputum purulence/volume; dyspnea; fever; hemoptysis; exercise intolerance; chest pain; increased pulmonary infiltration [6,14]. Exclusion criteria were antibiotics use within 4 weeks, malignancy, and failure to undergo measurements. To analyze discriminative performance in real-world settings, concomitant respiratory diseases (e.g. asthma) were not excluded.

Study protocol approval was obtained from Ethics Committee of The First Affiliated Hospital of Guangzhou Medical University. All patients gave written informed consent.

### Study design

The discriminative performance of LCI and RV/TLC%, and their association with clinical parameters of bronchiectasis were evaluated. Assessment included history inquiry, multiple-breath washout test, spirometry, and sputum culture. All baseline measurements were performed within 12 months of the chest HRCT assessment. To determine how ventilation heterogeneity or air trapping correlated with bronchodilator responses, we stratified according to LCI and RV/TLC (see details below). Our study design complied with the STROBE guideline (S1 Text).

### Clinical assessments

A radiologist with working experience of more than 10 years evaluated the CT scans and graded the radiological severity. Chest HRCT score was evaluated using modified Reiff score (0–6 points, mild bronchiectasis; 7–12 points, moderate bronchiectasis; 13–18 points, severe bronchiectasis) [6,14]. Dyshomogeneity (mosaicism), cystic bronchiectasis (denoted the presence of cystic changes of the dilated bronchi on chest HRCT), and the number of bronchiectatic lobes were evaluated.

A full description of the MBW is available in the Online Supplement text (S2 Text). MBW [6,15] with nitrogen as tracer gas was performed before spirometry, to derive the LCI values. Reference values of spirometry were derived from validated equations [16]. Following all MBW maneuvers which measured the functional residual volume, patients underwent slow vital capacity measurement in which an inspiratory and expiratory maneuver was involved, which was applied to calculate the total lung capacity and RV/TLC. The maximal values of forced vital capacity (FVC) and forced expiratory volume in one second (FEV_1_) were reported for spirometry. Bronchial dilation test was performed within the same day of LCI and RV/TLC measurement, in a subgroup of patients (concurrent bronchodilation test cohort) with FEV_1_ <80% predicted to minimize ceiling bronchodilator effects [14]. Inhaled long-acting beta-agonists or muscarinic receptor antagonists should be withheld for at least 12 hours, whereas short-acting beta-agonists or muscarinic receptor antagonists were withheld for at least 8 hours before bronchodilation test. Given small sample sizes, we also compared bronchodilator responses in patients who had ever undergone bronchodilation test within 2 years. Data were pooled (concurrent & previous bronchodilation test cohort) to increase statistical power of identifying different bronchodilator responses.

Following removal of oral debris, fresh sputum was obtained within 2 hours of sampling at initial visits. Bacterial (e.g. *Pseudomonas aeruginosa*) colonization was defined as two or more occasions (more than three months apart) with identical pathogenic bacteria being isolated from sputum within the nearest one year [16,17]. *Bronchiectasis Severity Index* (BSI) was employed to measure bronchiectasis severity, with 0–4, 5–8, and 9 points or greater corresponding to mild, moderate and severe bronchiectasis, respectively [17].

### Statistical analysis

Mean ± standard deviation or median (interquartile range) was expressed for numerical data, and compared with independent t-test or Mann-Whitney test. No. (%) was used for describing dichotomous data, and compared using chi-square tests.

LCI was normalized with RV/TLC (nLCI, calculated as dividing the LCI by RV/TLC) to assess whether it would improve discriminative performance of LCI. We adopted receiver operation characteristic (ROC) curve to compare how RV/TLC, LCI and nLCI discriminated moderate-to-severe from mild bronchiectasis, and mild-to-moderate from severe bronchiectasis according to BSI score. The area under curve (AUC), 95% confidence intervals (95%CI), sensitivity, specificity, accuracy and cut-off were calculated. The AUC was compared with MedCalc (MedCalc Inc., Ostend, Belgium). Chi-square test was applied to determine the consistency for dichotomous results of RV/TLC and LCI (lower and upper 50^th^ percentile). We assessed the correlation between RV/TLC and LCI, using partial correlation model with adjustment for age, sex, body-mass index and baseline FEV_1_% predicted.

We determined clinical variable attributes’ impacts on LCI, RV/TLC and nLCI using generalized linear mixed model, with factors of sex, sputum bacteriology (*Pseudomonas aeruginosa*/non-*Pseudomonas aeruginosa* colonized), cystic bronchiectasis (yes/no) and mosaicism (yes/no) as factors, and covariates of the number of bronchiectatic lobes, HRCT total score, age and baseline FEV_1_% predicted for fitting fixed-effect estimates.

To determine whether bronchodilator responses differ with ventilation heterogeneity and hyperinflation, RV/TLC (high vs. low) and LCI (high vs. low) were dichotomized according to their medians among all patients. Clinical characteristics of patients with higher RV/TLC and higher LCI (RV/TLC^High^LCI^High^), lower RV/TLC and higher LCI (RV/TLC^Low^LCI^High^), higher RV/TLC and lower LCI (RV/TLC^High^LCI^Low^), and lower RV/TLC and lower LCI (RV/TLC^Low^LCI^Low^) were compared. We did not adjust for multiple comparisons, therefore between-group comparisons were descriptive in nature.

Finally, we evaluated the correlation between bronchodilator responses (expressed either as improvement in FEV_1_ absolute values or percentage) and RV/TLC% and LCI.

Data analyses were processed with SPSS 16.0 package (SPSS Inc., Chicago, USA) and Graphpad Prism 5.0 (Graphpad Inc., San Diego, USA) unless otherwise stated. *P*<0.05 was deemed statistically significant.

## Results

### Subject enrollment

**Fig 1:**
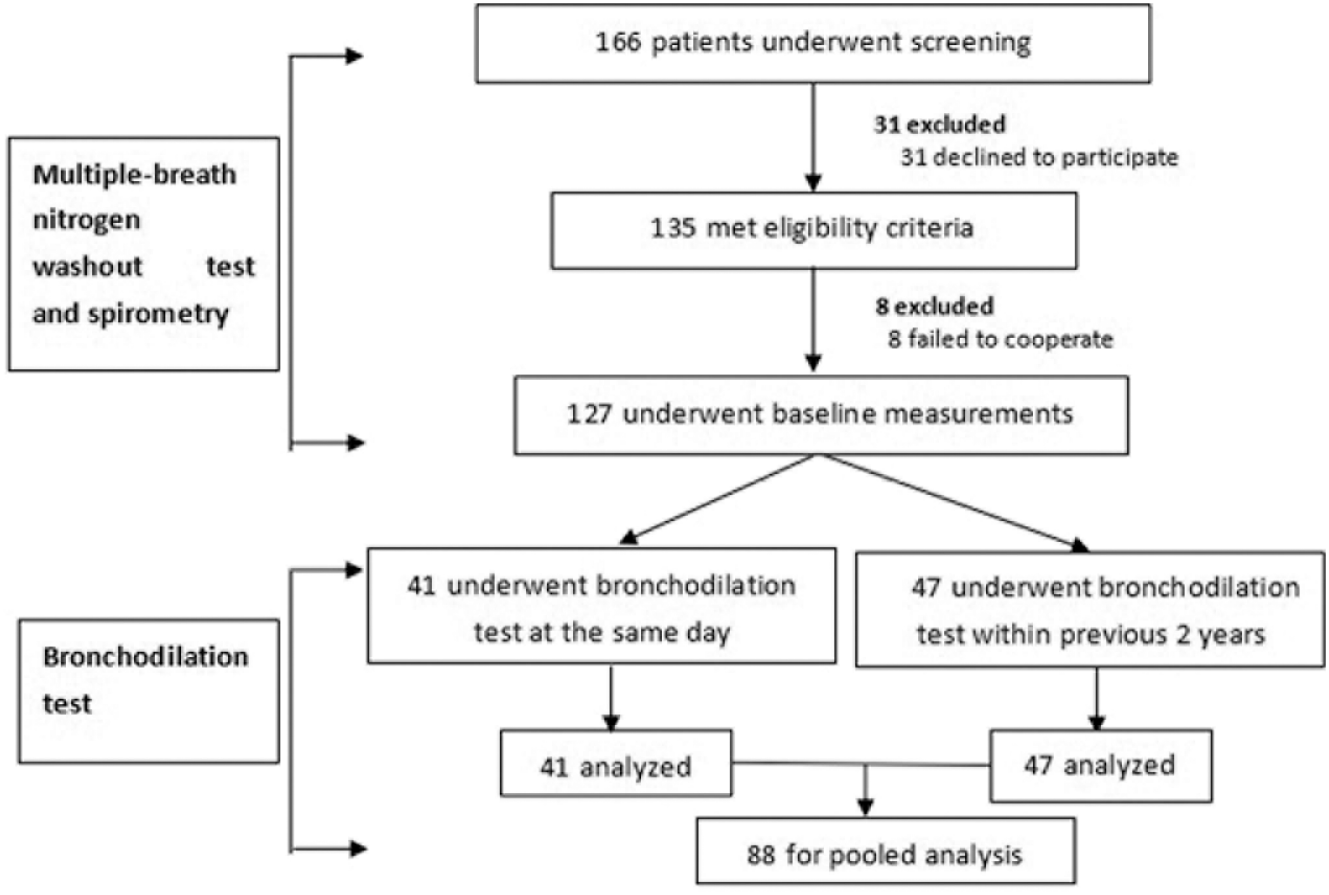
Recruitment flowchart. Of 166 patients who underwent screening, 127 were included in statistical analysis. 41 and 47 patients, respectively, had performed bronchodilation test within the same day, and in the previous 2 years, of LCI and RV/TLC measurement.

Data from 127 out of 166 patients who underwent screening were analyzed. 42 and 47 patients underwent bronchodilation test at baseline and at least once within 2 years, respectively (Fig 1). Bronchodilator response was evaluated in concurrent bronchodilation test cohort (n = 42) and pooled cohort (concurrent & previous bronchodilation test cohort, n = 88), respectively.

### Clinical characteristics

Of 127 bronchiectasis patients, most were female and never-smokers. The median BSI was 6.0. *Pseudomonas aeruginosa* was isolated from ~33% of cases. 85.8% of patients were never-smokers. 70% of patients had administered mucolytics within 6 months, but none was using inhaled antibiotics. Idiopathic and post-infectious constituted the most common etiologies. Asthma was deemed to be underlying causes in two patients (1.6%). Similar baseline characteristics were noted for concurrent and previous bronchodilation test cohorts, except for significant gender distribution. (Table 1)

**Table 1:** Clinical characteristics of bronchiectasis patients.

Parameter	All bronchiectasis patients (n = 127)	Concurrent bronchodilation test cohort (n = 41)^a^	Historic bronchodilation test cohort (n = 47)^b^	P value *
**Anthropometry**				
Age (years)	44.4±14.1	44.2±16.0	42.3±14.0	0.56
Females (No., %)	75 (59.1%)	16 (39.0%)	30 (63.8%)	0.02
BMI (kg/m^2^)	20.0 (18.1–23.2)	21.0±3.6	19.8±3.3	0.10
Never-smokers (No., %)	109 (85.8%)	31 (75.6%)	47 (100.0%)	<0.01
**History**				
No. of exacerbations within 2 years	3.0 (2.0–5.0)	3.0 (2.0–5.0)	3.0 (2.0–5.8)	0.87
**HRCT findings**				
No. of bronchiectatic lobes	4.0 (3.0–6.0)	5.0 (3.0–6.0)	5.0 (3.0–6.0)	0.56
HRCT total score	8.0 (5.0–11.0)	9.5±3.9	9.2±4.0	0.69
**Disease severity**				
*Bronchiectasis Severity Index*	6.0 (4.0–10.0)	7.2±3.5	7.4±3.5	0.81
**Sputum bacteriology**				
*Pseudomonas aeruginosa* (No., %)	42 (33.1%)	17 (41.5%)	18 (38.3%)	0.76
Other pathogenic bacteria (No., %) ^c^	40 (31.5%)	12 (29.3%)	17 (36.2%)	0.49
Commensals (No., %)	45 (35.4%)	11 (26.8%)	12 (25.5%)	0.89
**Medications within 6 months d**				
Inhaled corticosteroids (No., %)	33 (26.0%)	13 (31.7%)	13 (27.7%)	0.68
Mucolytics (No., %)	89 (70.1%)	25 (61.0%)	38 (80.9%)	0.04
Macrolides (No., %)	47 (37.0%)	17 (41.5%)	22 (46.8%)	0.61
**Underlying causese**				
Post-infectious (No., %)	39 (30.7%)	11 (26.8%)	15 (31.9%)	0.60
Miscellaneous known findings (No., %) ^d^^,^ ^e^	34 (26.8%)	10 (24.4%)	17 (36.2%)	0.23
Idiopathic (No., %)	56 (44.1%)	21 (51.2%)	17 (36.2%)	0.16
**Lung function parameters**				
LCI	14.6 (12.0–17.6)	16.2 (13.7–18.2)	16.4±4.0	0.94
RV/TLC	41.6±8.9	42.3±9.2	43.2±8.6	0.05

Numerical data were presented as either mean ± standard deviation (SD) or median (interquartile range, IQR) as appropriate. No patient was regularly using inhaled, oral or systemic antibiotics. LCI: lung clearance index; RV/TLC: the ratio of residual volume to total lung capacity

^a^ This cohort referred to the bronchiectasis patients who had undergone bronchodilation tests within the same day of multiple-breath nitrogen washout test and spirometry (also selected from the whole bronchiectasis cohort).

^b^ This cohort referred to the bronchiectasis patients who had undergone bronchodilation tests within the previous 2 years (also selected from the whole bronchiectasis cohort).

^c^ Other pathogenic bacteria for all bronchiectasis patients included *Haemophilus parainfluenzae* (n = 8, 7.3%), *Escherichia coli* (n = 5, 4.5%), *Klebsiella pneumoniae* (n = 4, 3.6%), *Serratia marcescens* (n = 2, 1.8%), *Streptococcus pneumoniae* (n = 1, 0.9%), *Moraxella catarrhalis* (n = 1, 0.9%), *Achromobacter xylosoxidans* (n = 1, 0.9%), *Proteus mirabilis* (n = 1, 0.9%), *Haemophilus haemolyticus* (n = 1, 0.9%) and *Bordetella bronchiseptica* (n = 1, 0.9%).

^d^ Most patients had ever used more than one category of medications within the last 6 months.

^e^ Dual underlying causes were determined in a minority of patients, thus the cumulative percentage was greater than 100%. Miscellaneous causes consisted of immunodeficiency, allergic bronchopulmonary aspergillosis, gastroesophageal reflux disease, asthma, diffuse panbronchiolitis, Kartagener syndrome, non-tuberculous mycobacteria disease, Young’s syndrome, rheumatoid arthritis, lung sequestration syndrome, and lung malformation.

* P values denoted the comparison between concurrent and historic bronchodilation test cohorts.

### The discriminative power of LCI, RV/TLC, and nLCI

Mean LCI and RV/TLC was 15.3 and 41.6%, with mean standard deviation of 0.83 and 1.70 for two technically acceptable measurements, respectively. Patients with either post-infectious or other etiologies had numerically higher LCI than those with idiopathic bronchiectasis; however, neither nLCI nor RV/TLC differ statistically when stratified by etiology (P>0.05, Table A in S2 Text).

**Fig 2:**
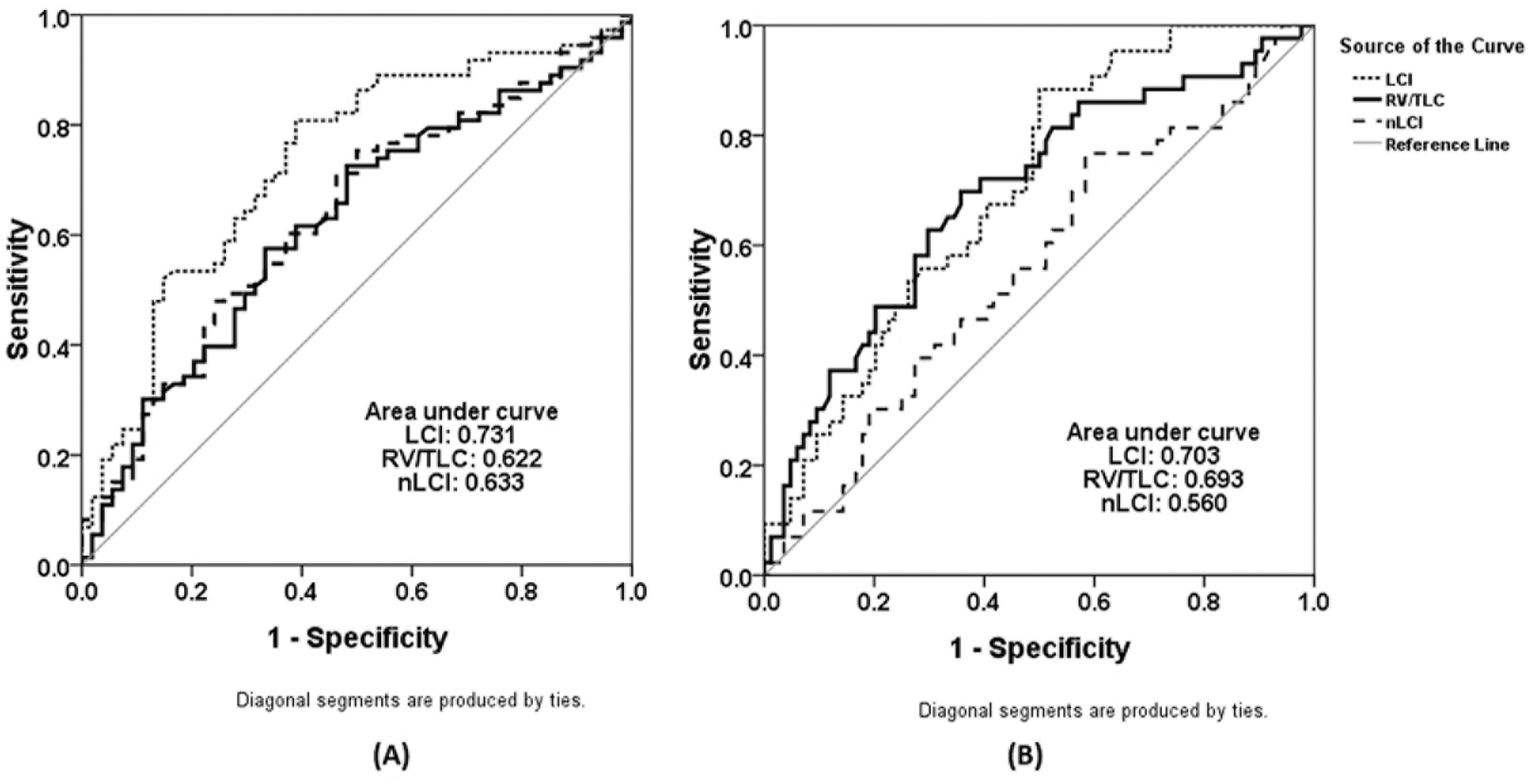
The discriminative performance of LCI, RV/TLC and nLCI in discriminating different subgroups of patients with bronchiectasis. (A). The discriminative performance of LCI, RV/TLC and nLCI for discriminating patients with moderate-to-severe bronchiectasis from those with mild bronchiectasis; AUC: 0.73, 95%CI: (0.64, 0.82) for LCI; AUC: 0.62, 95%CI: (0.52, 0.72) for RV/TLC, and AUC: 0.63, 95%CI: (0.54, 0.73) for nLCI; (B), The discriminative performance of LCI, RV/TLC and nLCI for discriminating patients with severe bronchiectasis from those with mild-to-moderate bronchiectasis; AUC: 0.70, 95%CI: (0.61, 0.79) for LCI; AUC: 0.69, 95%CI: (0.59, 0.79) for RV/TLC, and AUC: 0.56, 95%CI: (0.45, 0.67) for nLCI.

LCI (AUC: 0.73, 95%CI: 0.64, 0.82) demonstrated significantly greater diagnostic power (P = 0.038) than RV/TLC (AUC: 0.62, 95%CI: 0.52, 0.72) in discriminating moderate-to-severe from mild bronchiectasis (Fig 2-a and 2-b, Table B in S2 Text), whereas both parameters had comparable power in discriminating mild-to-moderate from severe bronchiectasis (AUC: 0.70 vs. 0.69, P = 0.839). The nLCI, however, did not confer additional discriminative performance compared with RV/TLC alone (P = 0.894) and was inferior to LCI (P = 0.020) in discriminating moderate-to-severe from mild bronchiectasis. Moreover, the nLCI had comparable discriminative performance with RV/TLC alone (P = 0.131) but was inferior to LCI (P<0.001) discriminating mild-to-moderate from severe bronchiectasis (Fig 2, Table B in S2 Text).

Moreover, LCI (AUC: 0.81, 95%CI: 0.73, 0.90) performed slightly better than RV/TLC (AUC: 0.72, 95%CI: 0.62, 0.82) in discriminating bronchiectasis patients with from without FEV_1_ >80% predicted (P = 0.129). Again, adopting the nLCI did not contribute to improved discriminative performance compared with LCI (P = 0.261 for nLCI vs. RV/TLC). (Table C in S2 Text)

### Consistency of LCI, RV/TLC and nLCI in reflecting clinical characteristics of bronchiectasis

Higher LCI was consistent with the clinical characteristics of bronchiectasis (all P<0.05) than RV/TLC in reflecting 3 or more bronchiectatic lobes (χ^2^: 31.69 vs. 14.38), HRCT total score of greater than 9 (χ^2^: 30.27 vs. 10.57), BSI of greater than 5 (χ^2^: 15.00 vs. 4.32), cystic bronchiectasis (χ^2^: 8.98 vs. 6.84), mosaicism (χ^2^: 15.01 vs. 9.29) and FEV_1_ ≤80% predicted (χ^2^: 21.17 vs. 11.47). However, normalization of LCI (nLCI) led to decreased consistency in reflecting 3 or more bronchiectatic lobes (χ^2^: 11.72, P<0.001), cystic bronchiectasis (χ^2^: 0.01, P = 0.952), mosaicism (χ^2^: 3.27, P = 0.071) and FEV_1_ predicted being not greater than 80% (χ^2^: 1.86, P = 0.173), except for HRCT total score being greater than 9 (χ^2^: 10.57, P = 0.001) and BSI being greater than 5 (χ^2^: 5.94, P = 0.015). (Table 2)

**Table 2:** Consistency of lung clearance index, the ratio of residual volume to total lung capacity, and normalized lung clearance index in reflecting the characteristics of clinical variables in clinically stable bronchiectasis.

	Higher LCI		Higher RV/TLC		Higher nLCI	
	χ^2^ statistics	P value	χ^2^ statistics	P value	χ^2^ statistics	P value
**>3 bronchiectatic lobes**	31.69	<0.001	14.38	0.001	11.72	<0.001
**HRCT total score >9**	30.27	<0.001	10.57	0.001	10.57	0.001
**BSI >5**	15.00	0.001	4.32	0.038	5.94	0.015
**Cystic bronchiectasis** *	8.98	0.003	6.84	0.009	0.01	0.952
**Mosaicism**	15.01	<0.001	9.29	0.002	3.27	0.071
**FEV**_**1**_ **predicted ≤80%**	21.17	<0.001	11.47	<0.001	1.86	0.173

Data are presented with counts unless otherwise stated. 95%CI: 95% confidence interval; BSI: *Bronchiectasis Severity Index*. LCI: lung clearance index; RV/TLC: the ratio of residual volume to total lung capacity; nLCI: normalized lung clearance index. Concordance was compared between the subgroups with higher or lower than the median of LCI, RV/TLC, and the normalized LCI, respectively. Because there was no “gold standard” for dichotomizing the HRCT score, we compared the data by using the median score of 9. For LCI, “Low” denoted the values being equal to or lower than the median (14.6), whereas “high” indicated the values being higher than the median (14.6). For RV/TLC, “Low” denoted the values being equal to or lower than the median (41.5%), whereas “high” indicated the values being higher than the median (41.5%). For normalized LCI, “Low” denoted the values being equal to or lower than the median (35.6), whereas “high” indicated the values being higher than the median (35.6). All median levels were derived from the whole bronchiectasis patient cohort (n = 127).

* Denoted the presence of cystic changes of the dilated bronchi on chest HRCT.

### Correlation between clinical parameters and LCI, RV/TLC, and nLCI

**Fig 3:**
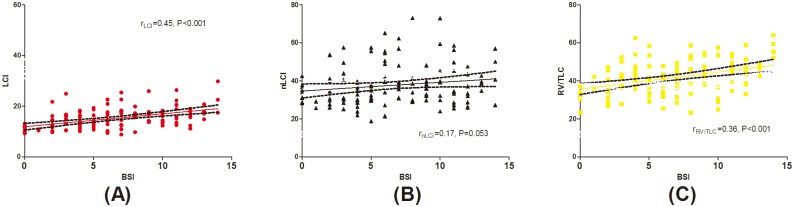
Correlation between the BSI and LCI, RV/TLC and nLCI in 127 patients with bronchiectasis when clinically stable. (A). Correlation between the BSI and LCI in patients with bronchiectasis. (B). Correlation between the BSI and the nLCI in patients with bronchiectasis. (C). Correlation between the BSI and RV/TLC in patients with bronchiectasis. The red dots represents LCI, the bright yellow rectangle represents RV/TLC, and the black triangle represents the nLCI. LCI: lung clearance index; RV/TLC: the ratio of residual volume to total lung capacity; nLCI: normalized lung clearance index.

Both LCI and RV/TLC increased progressively with greater BSI, with highest levels of LCI and RV/TLC in severe bronchiectasis. The BSI correlated significantly with LCI (r = 0.45, P<0.001) and RV/TLC (r = 0.36, P<0.001), but not the nLCI (r = 0.17, P = 0.053, Fig 3). In adjusted partial correlation model, LCI positively correlated with RV/TLC (r = 0.42, P<0.01). This applied in mild (r = 0.49, P<0.01) and severe bronchiectasis (r = 0.45, P<0.01), but not moderate bronchiectasis (r = 0.25, P = 0.12). (Table D in S2 Text) LCI was significantly higher in patients with RV/TLC >40% than those with RV/TLC ≤40% (S1 Fig). Moreover, in adjusted partial correlation model, FEV_1_/FVC% correlated with LCI (r = -0.48, P<0.01 in mild bronchiectasis; r = -0.62, P<0.01 in moderate bronchiectasis; r = -0.62, P<0.01 in severe bronchiectasis) and RV/TLC (r = -0.45, P<0.01 in mild bronchiectasis; r = -0.36, P = 0.02 in moderate bronchiectasis; r = -0.44, P<0.01 in severe bronchiectasis), but not nLCI (r = -0.20, P = 0.23 in mild bronchiectasis; r = -0.30, P = 0.06 in moderate bronchiectasis; r = -0.08, P = 0.64 in severe bronchiectasis; Table E in S2 Text). Nevertheless, we did not find significant correlation between functional residual capacity and RV/TLC (r = 0.09, P = 0.30).

Both LCI and RV/TLC significantly were associated with HRCT score, age, FEV_1_% predicted, and cystic bronchiectasis (all P<0.001). LCI was associated with *Pseudomonas aeruginosa* colonization, and had a borderline association with mosaicism; whereas RV/TLC correlated with gender. The nLCI had slightly greater effect sizes than LCI and RV/TLC in terms of correlation with HRCT score (estimate: 0.67, 95%CI: 0.57, 0.77), *Pseudomonas aeruginosa* colonization (estimate: -1.65, 95%CI: -2.09, -1.21), and cystic bronchiectasis (estimate: 3.28, 95%CI: 2.81, 3.74). (Table 3) Furthermore, subgroup analyses according to bronchiectasis severity showed similar variable attributes’ impacts on LCI, RV/TLC and nLCI (Tables F-H in S2 Text).

**Table 3:** Fixed-effect estimates in multivariate linear mixed model of the clinical variable attributes’ impacts on lung clearance index, the ratio of residual volume to total lung capacity, and normalized lung clearance index.

	LCI			RV/TLC			nLCI		
	Estimate	P value	95% CI	Estimate	P value	95% CI	Estimate	P value	95% CI
**Intercept**	**16.21**	**<0.001**	**14.98, 17.44**	**37.54**	**<0.001**	**36.31, 38.77**	**43.04**	**<0.001**	**41.81, 44.27**
**No. of bronchiectatic lobes**	-0.05	0.626	-0.66, 0.55	0.02	0.846	-0.19, 0.23	0.01	0.968	-0.21, 0.22
**HRCT total score** *	**0.39**	**<0.001**	**0.30, 0.49**	**0.31**	**<0.001**	**0.28, 0.30**	**0.67**	**<0.001**	**0.57, 0.77**
**Age**	**0.04**	**<0.001**	**0.03, 0.05**	**0.29**	**<0.001**	**0.28, 0.30**	**-0.18**	**<0.001**	**-0.20, -0.17**
**FEV**_**1**_ **predicted%**	**-0.09**	**<0.001**	**-0.10, -0.74**	**-0.19**	**<0.001**	**-0.20, -0.18**	**-0.03**	**<0.001**	**-0.04, -0.02**
**Sex**	-	-	-	-	-	-	-	-	-
Males	Reference	Reference	Reference	Reference	Reference	Reference	Reference	Reference	Reference
Females	0.14	0.475	-0.24, 0.51	**1.54**	**<0.001**	**1.17, 1.91**	**-0.86**	**<0.001**	**-1.23, -0.49**
***Pseudomonas aeruginosa* colonization**	-	-	-	-	-	-	-	-	-
Yes	Reference	Reference	Reference	Reference	Reference	Reference	Reference	Reference	Reference
No	**-0.85**	**<0.001**	**-1.29, -0.41**	0.17	0.441	-0.27, 0.61	**-1.65**	**<0.001**	**-2.09, -1.21**
**Cystic bronchiectasis** **	-	-	-	-	-	-	-	-	-
Yes	Reference	Reference	Reference	Reference	Reference	Reference	Reference	Reference	Reference
No	**1.11**	**<0.001**	**0.64, 1.57**	**-1.35**	**<0.001**	**-1.82, -0.89**	**3.28**	**<0.001**	**2.81, 3.74**
**Mosaicism**	-	-	-	-	-	-	-	-	-
Yes	Reference	Reference	Reference	Reference	Reference	Reference	Reference	Reference	Reference
No	-0.47	0.051	-0.95, 0.01	**-0.91**	**<0.001**	**-1.38, -0.44**	**-0.65**	**0.007**	**-1.12, -0.17**

95%CI: 95% confidence interval. LCI: lung clearance index; RV/TLC: the ratio of residual volume to total lung capacity; nLCI: normalized lung clearance index. Data in bold indicated the statistical analyses with significance.

* Modified Reiff score

** Denoted the presence of cystic changes of the dilated bronchi on chest HRCT.

### Clinical characteristics of bronchiectasis when stratified by LCI and RV/TLC

Table 4 shows the clinical characteristics (including bronchodilator response) among patients with higher or lower LCI and RV/TLC. Despite non-significant difference in symptom duration and prior exacerbation frequency, patients with higher LCI (LCI^High^RV/TLC^Low^ and LCI^High^RV/TLC^High^) had more bronchiectatic lobes, and higher HRCT score and BSI, and tended to have bilateral and cystic bronchiectasis, mosaicism, colonization by potentially pathogenic microorganisms (particularly *Pseudomonas aeruginosa*), and lower FEV_1_% compared with patients who had lower LCI (LCI^Low^RV/TLC^Low^ and LCI^Low^RV/TLC^High^). Despite non-significantly greater post-bronchodilator FEV_1_ in patients with LCI^High^RV/TLC^High^, differences in bronchodilator responses expressed either as absolute value or percentage change from baseline, were unremarkable among the four subgroups in both concurrent bronchodilation test cohort and the pooled cohort (S2 Fig). Furthermore, compared with patients in LCI^High^RV/TLC^Low^ subgroup, those with LCI^Low^RV/TLC^High^ were older and less likely to harbor *Pseudomonas aeruginosa*, and had greater FEV_1_. However, only numerically greater bronchodilator responses were demonstrated in patients with LCI^Low^RV/TLC^High^.

**Table 4:** Clinical characteristics of bronchiectasis patients stratified by LCI and RV/TLC levels.

Parameter	LCI^Low^RV/TLC^Low^ (n = 45)	LCI^Low^RV/TLC^High^ (n = 19)	LCI^High^RV/TLC^Low^(n = 19)	LCI^High^RV/TLC^High^ (n = 44)	P
**Anthropometry**					
Age (years)	41.6±13.2	52.9±13.1	36.5±12.2	48.0 (22.3)	**<0.001**
Females (No., %)	30 (66.7%)	10 (52.6%)	9 (47.4%)	25 (56.8%)	0.472
BMI (kg/m^2^)	20.9±3.3	20.9±3.8	20.0±2.8	20.3±3.7	0.716
**History**					
Duration of symptoms (yrs)	10.0 (16.0)	18.9±14.3	15.0 (9.0)	15.0 (18.3)	0.091
No. of exacerbations within the previous year	1.0 (2.0)	1.0 (1.0)	1.7±1.3	2.0 (2.0)	0.262
**HRCT findings**					
No. of bronchiectatic lobes	3.0 (2.0)	4.0 (3.0)	5.0 (2.0)	5.0 (2.0)	**<0.001**
HRCT total score	5.0 (4.5)	7.7±3.9	10.2±3.6	10.5±3.6	**<0.001**
Predominantly middle/lower lobe bronchiectasis	32 (71.1%)	10 (52.6%)	15 (78.9%)	28 (63.6%)	0.312
Bilateral bronchiectasis	29 (64.4%)	15 (78.9%)	19 (100.0%)	43 (97.7%)	**<0.001**
Cystic bronchiectasis *	23 (51.1%)	13 (68.4%)	14 (73.7%)	37 (84.1%)	**0.009**
Mosaicism	25 (55.6%)	14 (73.7%)	16 (84.2%)	41 (93.2%)	**<0.001**
**Disease severity**					
*Bronchiectasis Severity Index*	4.8±3.6	5.0 (6.0)	6.5±2.8	9.0 (6.0)	**<0.001**
**Sputum bacterial colonization**					
*Pseudomonas aeruginosa* (No., %)	7 (15.6%)	3 (15.8%)	6 (31.6%)	15 (34.1%)	**0.026**
Other pathogenic bacteria (No., %) ^a^	6 (13.3%)	1 (5.3%)	6 (31.6%)	19 (43.2%)	**<0.001**
**Lung function**					
FEV_1_% predicted	81.0±19.5	68.6±18.6	57.9±16.3	47.7±19.2	**<0.001**
Post-bronchodilator FEV_1_ change (L) ^b^	0.15±0.21	0.12±0.09	0.05±0.14	0.16±0.13	0.362
Post-bronchodilator FEV_1_ change (%) ^b^	7.9±14.4	7.0±5.2	3.4±8.1	12.1±8.5	0.171
Post-bronchodilator FEV_1_ change (L) ^c^	0.13±0.19	0.13±0.11	0.11±0.14	0.14±0.11	0.883
Post-bronchodilator FEV_1_ change (%) ^c^	8.1±15.1	6.5 (8.8)	8.1±10.6	11.5±8.8	0.163

For LCI, “Low” denoted the values being equal to or lower than the median (14.6), whereas “high” indicated the values being higher than the median (14.6). For RV/TLC%, “Low” denoted the values being equal to or lower than the median (41.5%), whereas “high” indicated the values being higher than the median (41.5%). Significant bronchodilator response was defined as post-bronchodilator FEV_1_ increased by at least 12% and 200 ml. The P value demonstrated inside the table referred to the comparison among the four groups.

* Denoted the presence of cystic changes of the dilated bronchi on chest HRCT.

^a^ Other colonized bacteria in this study included *Haemophilus parainfluenzae*, *Escherichia coli*, *Klebsiella pneumoniae*, *Serratia marcescens*, *Streptococcus pneumoniae*, *Moraxella catarrhalis*, *Achromobacter xylosoxidans*, *Proteus mirabilis*, *Haemophilus haemolyticus* and *Bordetella bronchiseptica*.

^b^ Statistical analyses were done in 7, 8, 7 and 19 patients with available data (bronchodilator tests performed within the same day of the multiple-breath washout test) in groups LCI^Low^RV/TLC^Low^, LCI^Low^RV/TLC^High^, LCI^High^RV/TLC^Low^, and LCI^High^RV/TLC^High^, respectively (Concurrent bronchial dilation test cohort).

^c^ Statistical analyses were done in 19, 13, 16 and 40 patients with available data in groups LCI^Low^RV/TLC^Low^, LCI^Low^RV/TLC^High^, LCI^High^RV/TLC^Low^, and LCI^High^RV/TLC^High^, respectively. Results for patients who performed bronchilation test at baseline visits and those who had performed within the previous 2 years were pooled for analysis (Concurrent & historic bronchial dilation test cohort).

## Discussion

### Main findings

MBW are simple and feasible for measuring ventilation heterogeneity and the functional residual volume. Despite growing application of LCI for bronchiectasis assessment, no study has mentioned the potential impacts of RV/TLC on analysis of LCI. Conventionally, raw LCI values have been consistently reported. This study comprehensively compared the discriminative performance of LCI and RV/TLC in bronchiectasis across the whole severity spectrum, and compared with the normalized parameter (nLCI). Furthermore, we compared bronchodilator responses by stratifying the LCI and RV/TLC. Overall, our findings justify the report of raw, but not normalized, values of LCI.

### Interpretation

Historically, head-to-head comparisons of LCI and RV/TLC are lacking. The positive correlations between them might be primarily attributable to more aberrant airway architectures associated with greater disease severity. Findings from CF showed that LCI abnormality (84%) was more common than lung hyperinflation (58%) measured with residual volume in young children [18], and that LCI was more sensitive than functional residual capacity to discriminate *Pseudomonas aeruginosa* plus other pathogenic bacteria infection in children [19]. However, in the study by Gustafsson et al [20], LCI had greater sensitivity to detect abnormal lung structure which incorporated air trapping >30% and bronchiectasis in CF. Our study was the first to document that LCI conferred superior discriminative performance to RV/TLC for bronchiectasis assessment. Theoretically, gas trapping leading to hyperinflation is the consequence of increased bronchial wall thickness and decreased attenuation [21] which are associated with airway remodeling following recurrent infection and inflammation; whereas LCI correlated with airway mucus plugging (which had no significant impacts on airway obstruction) [21] and bronchial wall thickness [22]. Discordant changes in lung volume and LCI have been shown in bronchiectasis following chest physiotherapy which sought to mitigate mucus plugging [23]. Therefore, LCI might have greater discriminative performance and better correlation with clinical variables of bronchiectasis than hyperinflation indices such as RV/TLC.

Normalization of LCI with RV/TLC did not contribute to improved discriminative performance or correlation with the clinical variables of bronchiectasis compared with either parameter alone. This indicated that the magnitude of hyperinflation did not significantly affect ventilation heterogeneity. In the study by Haidopoulou et al., adjustment of LCI with airway and/or equipment dead space did not improve the sensitivity in identifying early CF lung disease (which frequently included bronchiectasis) in children [24]. Thus, LCI was virtually unaffected by dead space volume, either from the respiratory tract or testing equipment. One might argue the numerically greater correlation of nLCI with clinical parameters; however, the priority of clinical relevance should be given to discriminative performance and concordance with other clinical variables. Our findings remained unchanged following stratification of bronchiectasis severity, suggesting that disease severity might not be a modifier of the relationship between LCI and RV/TLC. Therefore, the additive value of normalization with RV/TLC is minimal.

Because of airway remodeling and mucus plugging, ventilation heterogeneity and hyperinflation might be reduced following bronchodilation. Unexpectedly, stratifying the LCI and RV/TLC did not reveal different bronchodilator responses. Recent literature suggested that RV more sensitively reflected airway reversibility than spirometric parameters (including FEV_1_) in obstructive lung disease [25], and that hyperinflation was associated with greater bronchodilator responses in patients with chronic obstructive pulmonary disease who had poorer lung function [26]. These collectively indicated the potential significance of hyperinflation indices (including RV/TLC) as markers of bronchodilator responses. Contrarily, documentation of different bronchodilator responses when stratified by LCI has not been available. In our study, patients with consistently higher LCI and RV/TLC showed greater disease severity and numerically greater bronchodilator responses, which might have approached statistical significance if sample sizes were further increased. Nevertheless, when specifically focusing on LCI^High^RV/TLC^Low^ and LCI^Low^RV/TLC^High^ subgroup, only numerically greater bronchodilator responses were shown in the latter subgroup. However, when analyzing previous records (within 2 years), this trend was slightly tempered. Due to limited sample sizes, we cannot justify the additive value of RV/TLC to LCI in identifying different bronchodilator responses.

### Limitations

Our findings are limited by the sample sizes (particularly bronchodilator response) from single research center. No longitudinal follow-up was scheduled to determine temporal stability of LCI or RV/TLC. We did not stratify the levels of LCI based on the previously published “normal values” or the cut-off values derived from receiver operation characteristic curve because all patients in this study demonstrated increased LCI and no healthy subject was enrolled. In light of the “gold standard” for classification, we think that the stratification based on the median levels among all bronchiectasis patients remained to be the alternative approach for classification. The use of MBW for the measurement of LCI might have limited value particularly among patients with severe airflow obstruction or emphysema, in whom the true value of functional residual volume might have been underestimated by the washout methods. Specifically, MBW does not take into account the trapped gas or completely obstructed lung zones. In this study, there lacks a validation of the functional residual capacity using the same pulmonary function testing instrument for LCI measurement. It should be recognized that body plethysmography remains the gold standard for measuring RV/TLC. However, for ease of patient’s cooperation, body plethysmography was not additionally performed. The MBW method used in our study has limitations (not validated in this population). There was an absence of age-matched normative data at our local site. We only utilized simple normalization algorithms for adjusting LCI, based on the *priori* assumption that ventilation heterogeneity could be affected by the magnitude of hyperinflation. Nevertheless, such an attempt remains flawed because nLCI could be simultaneously influenced by different levels of LCI and RV/TLC, for instance, lower nLCI could be derived from lower LCI plus higher RV/TLC which might be contradictory to our research hypothesis. We cannot fully exclude other more sensitive parameters aside from LCI. However, findings from Haidopoulou et al. have minimized the possibility of deriving more sensitive indices [24]. Additionally, there still might be a bias in the influence of bronchodilation response because it was not assessed in all those with FEV_1_ >80%. Finally, the criterion for defining exacerbations was not based on the latest expert consensus, which might have affected some of the data interpretation.

### Clinical implications

LCI is a sensitive marker for assessment of bronchiectasis [3], primary ciliary dyskinesia [27] and CF [20]. From physiological points of view, potential confounding of LCI by RV/TLC cannot be thoroughly excluded. Importantly, our comprehensive analysis has justified the reporting of raw values of LCI against further attempts to normalize with RV/TLC in bronchiectasis. For clinicians, LCI can be reported without further needs to incorporate the levels of RV/TLC. In other words, RV/TLC confers no significant additional benefits for ventilation heterogeneity measurement with LCI. Caution should be exercised when predicting bronchodilator responses with LCI in combination of RV/TLC.

## Supporting information

S1 File RV TLC LCI.xls. This is an elementary electronic data base with information pertinent to the clinical information and lung function assessment in patients with bronchiectasis.
(XLS)

S1 Text STROBE checklist. (DOC)

S2 Text (DOC)

S1 Fig Comparison of LCI in patients with or without RV/TLC >0.40. (TIF)

S2 Fig Difference between pre- and post-bronchodilator FEV_1_ in patients with bronchiectasis. (A). Difference between pre- and post-bronchodilator FEV_1_ (expressed in absolute values) in bronchiectasis patients who underwent bronchodilation test within the same day of multiple-breath nitrogen washout test and spirometry; Statistical analyses were done in 7, 8, 7 and 19 patients with available data (bronchodilator tests performed within the same day of the multiple-breath washout test) in groups LCI^Low^RV/TLC^Low^, LCI^Low^RV/TLC^High^, LCI^High^RV/TLC^Low^, and LCI^High^RV/TLC^High^, respectively. Overall, no significant difference was observed when comparing the data among the four subgroups. (B). Difference between pre- and post-bronchodilator FEV_1_ (expressed in percentage) in bronchiectasis patients who underwent bronchodilation test within the same day of multiple-breath nitrogen washout test and spirometry; Statistical analyses were done in 7, 8, 7 and 19 patients with available data (bronchodilator tests performed within the same day of the multiple-breath washout test) in groups LCI^Low^RV/TLC^Low^, LCI^Low^RV/TLC^High^, LCI^High^RV/TLC^Low^, and LCI^High^RV/TLC^High^, respectively. Overall, no significant difference was observed when comparing the data among the four subgroups. (C). Difference between pre- and post-bronchodilator FEV_1_ (expressed in absolute values) in all bronchiectasis patients who had ever undergone bronchodilation test (either at the baseline visit or within the previous 2 years); Statistical analyses were done in 19, 13, 16 and 40 patients with available data (bronchodilator tests performed within the same day of the multiple-breath washout test) in groups LCI^Low^RV/TLC^Low^, LCI^Low^RV/TLC^High^, LCI^High^RV/TLC^Low^, and LCI^High^RV/TLC^High^, respectively. Overall, no significant difference was observed when comparing the data among the four subgroups. (D). Difference between pre- and post-bronchodilator FEV_1_ (expressed in percentage) in all bronchiectasis patients who had ever undergone bronchodilation test (either at the baseline visit or within the previous 2 years); Statistical analyses were done in 19, 13, 16 and 40 patients with available data (bronchodilator tests performed within the same day of the multiple-breath washout test) in groups LCI^Low^RV/TLC^Low^, LCI^Low^RV/TLC^High^, LCI^High^RV/TLC^Low^, and LCI^High^RV/TLC^High^, respectively. Overall, no significant difference was observed when comparing the data among the four subgroups.
(TIF)

## Abbreviations

95%CI: 95% confidence interval
AUC: area under curve
BSI: *Bronchiectasis Severity Index*
CF: cystic fibrosis
FEV_1_: forced expiratory volume in one second
FVC: forced vital capacity
HRCT: high-resolution computed tomography
LCI: lung clearance index
MBW: multiple-breath nitrogen washout tests
nLCI: normalized lung clearance index
ROC: receiver operation characteristic
RV/TLC: the ratio of residual volume to total lung capacity
