# Supplementary material for: Residual volume/total lung capacity ratio confers limited additive significance to lung clearance index for assessment of adults with bronchiectasis
Source: PLoS One. 2017 Sep 8;12(9):e0183779. doi: 10.1371/journal.pone.0183779 (PMC5590849; doi:10.1371/journal.pone.0183779)
Supplement: S2 Text — (DOC) [file pone.0183779.s003.doc]

**Online supplement**

**Residual volume/total lung capacity ratio confers limited additive significance to lung clearance index for assessment of adults with bronchiectasis**

**Wei-jie Guan ^1,2^, Ph.D., Jing-jing Yuan ^1^, M.Sc., Yan Huang ^1^, M. Med., Hui-min Li ^1^, M.T., Rong-chang Chen ^1^, M.D., Nan-shan Zhong ^1^, M.D.**

^1^ State Key Laboratory of Respiratory Disease, National Clinical Research Center for Respiratory Disease, Guangzhou Institute of Respiratory Disease, First Affiliated Hospital of Guangzhou Medical University, Guangzhou, Guangdong, China

^2^ Sino-French Hoffmann Institute, Guangzhou Medical University, Guangzhou, China

**Corresponding author 1:** Rong-chang Chen, M. D., State Key Laboratory of Respiratory Disease, National Clinical Research Center for Respiratory Disease, Guangzhou Institute of Respiratory Disease, First Affiliated Hospital of Guangzhou Medical University, Address: 151 Yanjiang Road, Guangzhou, Guangdong, China, Fax: +86-20-83062719, Phone: +86-20-83062719, E-mail: [chenrc@vip.163.com](mailto:chenrc@vip.163.com)

**Corresponding author 2:** Wei-jie Guan, Ph. D., State Key Laboratory of Respiratory Disease, National Clinical Research Center for Respiratory Disease, Guangzhou Institute of Respiratory Disease, First Affiliated Hospital of Guangzhou Medical University, Address: 151 Yanjiang Road, Guangzhou, Guangdong, China, Fax: +86-20-83062719, Phone: +86-20-83062876, E-mail: [battery203@.163.com](mailto:battery203@.163.com)

**E-mail address for all authors**

Dr. Wei-jie Guan: [battery203@163.com](mailto:battery203@163.com)

Ms Jing-jing Yuan: [jacyyjj@163.com](mailto:jacyyjj@163.com)

Ms. Yan Huang: dr_hyan324@163.com

Ms Hui-min Li: [905488325@qq.com](mailto:905488325@qq.com)

Prof. Rong-chang Chen: [chenrc@vip.163.com](mailto:chenrc@vip.163.com)

Prof. Nan-shan Zhong: [nanshan@vip.163.com](mailto:nanshan@vip.163.com)

**Author contributions:** W. J. G. drafted the manuscript; J. J. Y., R. C. C. and N. S. Z. were responsible for patient recruitment; W. J. G., H. M. L., Y. H. and J. J. Y. collected individual data; W. J. G. performed statistical analyses; W. J. G. contributed to study conception; R. C. C. and N. S. Z. reviewed the manuscript and approved the final submission.

**Source of funding:** National Natural Science Foundation No. 81400010, Pearl River S&T Nova Program of Guangzhou No. 201710010097 and Guangdong Province Universities and Colleges Pearl River Scholar Funded Scheme 2017 (to Dr. Guan), Changjiang Scholars and Innovative Research Team in University ITR0961, The National Key Technology R&D Program of the 12th National Five-year Development Plan 2012BAI05B01 and National Key Scientific & Technology Support Program: Collaborative innovation of Clinical Research for chronic obstructive pulmonary disease and lung cancer No. 2013BAI09B09 (to Profs. Zhong and Chen).

**Conflict of interest:** Dr. Guan declared that he has received National Natural Science Foundation No. 81400010, Pearl River S&T Nova Program of Guangzhou No. 201710010097 and Guangdong Province Universities and Colleges Pearl River Scholar Funded Scheme 2017. Profs. Zhong and Chen declared that they had received Changjiang Scholars and Innovative Research Team in University ITR0961, The National Key Technology R&D Program of the 12th National Five-year Development Plan 2012BAI05B01 and National Key Scientific & Technology Support Program: Collaborative innovation of Clinical Research for chronic obstructive pulmonary disease and lung cancer No. 2013BAI09B09. All other authors declared no potential conflict of interest. None of the funding sources had any role on the study.

Data are from the Guangzhou Bronchiectasis Study whose authors may be contacted with Dr. Wei-jie Guan ([battery203@163.com](mailto:battery203@163.com)). This does not alter our adherence to PLOS ONE policies on sharing data and materials.

**Methods**

**Multiple-breath nitrogen washout test (MBW)**

Lung clearance index (LCI) and the ratio of residual volume and total lung capacity (RV/TLC) were measured prior to spirometry, with the multiple-breath nitrogen washout technique, by using the validated QUARK PFT real-time gas-analyzer (COSMED Inc., Italy) which has been employed for our routine clinical practice. The instrument has been calibrated to ensure the assay accuracy each day, prior to the measurement. The accuracy of the gas analyzer measuring nitrogen concentration was within 1% relative of the full scale, according to the manufacturer’s specification.

Before measurement, a designated, experienced research technician was responsible for interpretation of the main purposes and procedures of MBW test. Patients were seated with a nose clip applied, and calmly breathed in pure oxygen gas (oxygen concentration 99.5% or greater) from the open circuit through the mouthpiece whilst avoiding gas leakage. Patients were requested to maintain a steady respiratory rate of 12-16 breaths per minute, with the breathing volume of approximately 1.0 L (which can be graphically displayed on the computer screen, in a real-time fashion, at the pre-phase of and during the MBW test). Artifacts, such as cough, breathe with irregular small volumes, evidence of significant trapped gas with larger breaths, or glottis closure, should also be avoided throughout the measurement. We discarded any test with the above-mentioned artifacts. The proper maneuvers were repeated until the exhaled nitrogen concentration reached to 1/40^th^ of the original concentration (typically 2.5%) or lower. At least two measurements at 10-minute intervals (which exceeded the single washout time to allow for nitrogen concentration to return to baseline levels) were performed, which enabled the calculation of the mean LCI and functional residual volume. The residual volume (RV) could be subsequently derived from the functional residual volume, which was calculated with the formula as described below:

| FRC = | (V_E_ - V_DS_) - (C_F_ - C_S_) -V_TIS_ |
| --- | --- |
|  | C_AI_ - C_AF_ |

FRC: functional residual capacity; V_E_: the expired gas volume; V_DS_: dead space volume of the instrument; C_F_: the concentration of nitrogen within the testing tube; C_S_: the nitrogen concentration within the oxygen tank; V_TIS_: the volume of nitrogen being exhaled during the test; C_AI_: the nitrogen concentration within the alveoli, before oxygen inhalation; C_AF_: the nitrogen concentration within the alveoli, after completing the measurement.

We discarded any maneuver in case the difference in functional residual capacity was greater than 10%, evidence of gas leakage or irregular breathing [E1, E2].

LCI denoted the number of lung volume turnovers (cumulative expired volume divided by functional residual capacity) which entailed the reduction in end-expiratory nitrogen concentration to 1/40^th^ of its initial concentration. Higher LCI denoted a greater magnitude of ventilation heterogeneity.

MBW with nitrogen as tracer gas was performed before spirometry, to calculate the LCI values. Reference values of spirometry were derived from validated equations. Following all MBW maneuvers, patients underwent slow vital capacity measurement in which an inspiratory and expiratory maneuver was involved, which was applied to calculate the total lung capacity (TLC) and the RV/TLC.

**Spirometry**

We performed spirometry using spirometers (QUARK PFT, COSMED Inc., Italy) based on international guidelines. Results were derived from 3 technically repeatable maneuvers, with between-maneuver variation <5% or 200ml in forced vital capacity (FVC) and FEV_1_. Maximal FVC and FEV_1_ were reported. Predicted values were recommended by Zheng et al.

**Bronchodilator test**

Bronchial dilation test was performed in patients with FEV_1_ predicted less than 80%. Salbutamol (GlaxoSmithKline Inc., UK) 400 micrograms was administered via spacer (Volumatic & Handbury’s, UK). This entailed spirometry reassessment at 15 minutes post-bronchodilation. BDR was expressed as either absolute or percentage of FEV_1_ improvement following salbutamol inhalation.

In this study, bronchodilator response was evaluated in concurrent bronchodilation test cohort (n=42) and the pooled cohort (n=88), respectively. (**Table 1, Fig 1**)

**Study cohorts**

Bronchial dilation test was performed within the same day of LCI and RV/TLC measurement, in a subgroup of patients (concurrent bronchial dilation test cohort) with FEV_1_ less than 80% predicted to minimize ceiling bronchodilator effects. Given the relatively small sample size, we also compared the bronchodilator responses in bronchiectasis patient who had ever undergone bronchial dilation test in our research center within 2 years. Data were pooled (concurrent & historical bronchial dilation test cohort) to further increase the statistical power of identifying different bronchodilator responses.

**Sputum bacteriology**

Fresh sputum was sampled during hospital visits. Following removal of debris in oral cavity, patients expectorated into sterile container for bacterial culture. Hypertonic saline (3%~5%) induction, previously validated, was applied as appropriate. Sputum was sent for bacterial culture within 2 hours of sampling.

Blood and chocolate agar plates (Biomeurix, France) were adopted as culture media. Fresh sputum was homogenized with SPUTASOL (Oxoid SR089A, Cambridge, UK) and serially diluted with natural saline at concentrations of 10^-4^, 10^-5^ and 10^-6^. This was followed by addition of 10μl respective diluent to the plates with micropipette tube and inoculation using 10μl standardized rings. Plates were positioned in thermostatic box containing 5% carbon dioxide at 37℃ for overnight incubation.

*Pseudomonas aeruginosa* colonization denoted sputum culture positive for 2 or more occasions (at least 3 months apart) within 1 year.

***Bronchiectasis Severity Index***

*Bronchiectasis Severity Index* (BSI) was used to evaluate disease severity. The BSI was an integrated disease severity metric that comprised of the age, body-mass index, the number of bronchiectasis exacerbation and hospitalization for exacerbation in the previous one year, Medical Research Council dyspnea score, FEV_1_ predicted%, *P. aeruginosa* colonization, colonization with other PPMs and the number of bronchiectatic lobes, The established cut-off value of 0-4, 5-8, and 9 or greater corresponded to mild, moderate and severe bronchiectasis, respectively.

**Radiologic assessment**

We utilized the modified Reiff score for assessment of radiologic severity of bronchiectasis. The HRCT score was assessed on a lobar basis (lingular lobe as a separate lobe). For individual lobes, the extent of bronchiectasis was scored (0 for no, 1 for tubular, 2 for varicose and 3 for cystic bronchiectasis). Total HRCT score was derived by summing the score of 6 lung lobes (maximal total score: 18). We classified the HRCT score into tertiles: 0-6 for mild bronchiectasis, 7-12 for moderate bronchiectasis, and 13-18 for severe bronchiectasis.

**Reference**

E1. Rowan SA, Bradley JM, Bradbury I, et al. Lung clearance index is a repeatable and sensitive indicator of radiological changes in bronchiectasis. Am J Respir Crit Care Med. 2014; 189: 586-92

E2. Grillo L, Irving S, Hansell DM, et al. The reproducibility and responsiveness of the lung clearance index in bronchiectasis. Eur Respir J. 2015; 46: 1645-53

**RESULTS**

**Discriminative performance**

Mean LCI and RV/TLC was 15.3 and 41.6%, with the mean standard deviation of 0.83 and 1.70 for two technically acceptable measurements, respectively. Patients with either post-infectious or other etiologies had numerically higher LCI than those with idiopathic bronchiectasis; however, neither nLCI nor RV/TLC differ statistically when stratified by etiology (P>0.05, **Table A in S2 Text**).

LCI (AUC: 0.73, 95%CI: 0.64, 0.82) demonstrated significantly greater diagnostic power (P=0.038) than RV/TLC (AUC: 0.62, 95%CI: 0.52, 0.72) in discriminating moderate-to-severe from mild bronchiectasis (**Fig 2, Table B in S2 Text**), whereas both parameters had comparable power in discriminating mild-to-moderate from severe bronchiectasis (AUC: 0.70 vs. 0.69, P=0.839). The nLCI, however, did not confer additional discriminative performance compared with RV/TLC alone (P=0.894) and was inferior to LCI (P=0.020) in discriminating moderate-to-severe from mild bronchiectasis. Moreover, the nLCI had comparable discriminative performance with RV/TLC alone (P=0.131) but was inferior to LCI (P<0.001) discriminating mild-to-moderate from severe bronchiectasis (**Fig 2**, **Table A in S2 Text**).

LCI (AUC: 0.81, 95%CI: 0.73, 0.90) performed slightly better than RV/TLC (AUC: 0.72, 95%CI: 0.62, 0.82) in discriminating bronchiectasis patients with and without FEV_1_ >80% predicted (P=0.129). Again, adopting the nLCI did not contribute to improved discriminative performance (P=0.261 for nLCI vs. RV/TLC). (**Table C in S2 Text**)

**Correlation between LCI and RV/TLC**

Both LCI and RV/TLC increased progressively with greater BSI, with highest levels of LCI and RV/TLC in severe bronchiectasis. (**Table D in S2 Text**) The BSI correlated significantly with LCI (r=0.45, P<0.001) and RV/TLC (r=0.36, P<0.001), but not the nLCI (r=0.17, P=0.053, **Fig 3**). Using adjusted partial correlation model, we found that LCI positively correlated with RV/TLC (r= 0.42, P<0.01). This finding applied in mild (r= 0.49, P<0.01) and severe bronchiectasis (r= 0.45, P<0.01), but not moderate bronchiectasis (r= 0.25, P=0.12). (**Table C in S2 Text**) Moreover, LCI was significantly higher in patients with RV/TLC >40% than those with RV/TLC ≤40% (**S1 Fig**).

In adjusted partial correlation model, FEV_1_/FVC% correlated with LCI (r=-0.48, P<0.01 in mild bronchiectasis; r=-0.62, P<0.01 in moderate bronchiectasis; r=-0.62, P<0.01 in severe bronchiectasis) and RV/TLC (r=-0.45, P<0.01 in mild bronchiectasis; r=-0.36, P=0.02 in moderate bronchiectasis; r=-0.44, P<0.01 in severe bronchiectasis), but not nLCI (r=-0.20, P=0.23 in mild bronchiectasis; r=-0.30, P=0.06 in moderate bronchiectasis; r=-0.08, P=0.64 in severe bronchiectasis; **Table E in S2 Text**). Nevertheless, we did not find significant correlation between functional residual capacity and RV/TLC (r=0.09, P=0.30).

**LCI had greater consistency in reflecting clinical characteristics of bronchiectasis**

Higher levels of LCI had consistently greater consistency (all P<0.05) than RV/TLC in reflecting 3 or more bronchiectatic lobes (χ^2^: 31.69 vs. 14.38), HRCT total score of greater than (χ^2^: 30.27 vs. 10.57), BSI of greater than 5 (χ^2^: 15.00 vs. 4.32), cystic bronchiectasis (χ^2^: 8.98 vs. 6.84), mosaicism (χ^2^: 15.01 vs. 9.29) and FEV_1_ ≤80% predicted (χ^2^: 21.17 vs. 11.47). However, normalization of LCI (nLCI) led to decreased consistency in reflecting 3 or more bronchiectatic lobes (χ^2^: 11.72, P<0.001), cystic bronchiectasis (χ^2^: 0.01, P=0.952), mosaicism (χ^2^: 3.27, P=0.071) and FEV_1_ predicted being not greater than 80% (χ^2^: 1.86, P=0.173), except for HRCT total score being greater than 9 (χ^2^: 10.57, P=0.001) and BSI being greater than 5 (χ^2^: 5.94, P=0.015). (**Table 2**)

**Clinical variable attributes’ impacts on LCI, RV/TLC and nLCI**

LCI and RV/TLC significantly correlated with HRCT total score, age, FEV_1_% predicted, and presence of cystic bronchiectasis (all P<0.001). LCI correlated with *Pseudomonas aeruginosa* colonization, and had a borderline correlation with mosaicism; whereas RV/TLC also correlated with gender. The nLCI had greater effect sizes than LCI and RV/TLC in terms of HRCT total score (estimate: 0.67, 95%CI: 0.57, 0.77), *Pseudomonas aeruginosa* colonization (estimate: -1.65, 95%CI: -2.09, -1.21), and cystic bronchiectasis (estimate: 3.28, 95%CI: 2.81, 3.74). (**Table 3**)

Subgroup analyses according to the BSI (BSI <5, 5≤BSI<9, and BSI≥9) showed similar despite varying findings on clinical variable attributes’ impacts on LCI, RV/TLC and nLCI (**Tables F-H in S2 Text**).

**Clinical characteristics among patients with higher or lower levels of LCI and RV/TLC**

Despite the lack of difference in symptom duration and prior exacerbation frequency, patients with higher LCI (LCI^High^RV/TLC^Low^ and LCI^High^RV/TLC^High^) had more bronchiectatic lobes, and higher HRCT total score and BSI, and tended to have bilateral and cystic bronchiectasis, ventilation heterogeneitymosaicism, colonization by potentially pathogenic microorganisms (particularly *Pseudomonas aeruginosa*), and lower FEV_1_% compared with patients who had lower LCI (LCI^Low^RV/TLC^Low^ and LCI^Low^RV/TLC^High^). Despite a trend towards greater post-bronchodilator FEV_1_ in patients with LCI^High^RV/TLC^High^, the difference in bronchodilator responses expressed either as the absolute value or percentage change from baseline, were unremarkable among the four subgroups in both concurrent bronchial dilation test cohort and the pooled cohort (**S2 Fig in S2 Text**). Furthermore, compared with patients in LCI^High^RV/TLC^Low^ subgroup, those with LCI^Low^RV/TLC^High^ were older and less likely to harbor *Pseudomonas aeruginosa,* and had greater FEV_1_. However, only numerically greater bronchodilator responses were demonstrated in patients with LCI^Low^RV/TLC^High^.

**Table A Comparison of LCI, normalized LCI, and RV/TLC according to bronchiectasis etiology**

|  | **Post-infectious** | **Other known etiologies** | **Idiopathic** | **P value** |
| --- | --- | --- | --- | --- |
| **LCI** | 15.9±3.9 | 16.0±4.7 | 13.3 (5.3) | 0.047 |
| **nLCI** | 34.9 (14.1) | 38.2 (12.4) | 34.4 (11.8) | 0.516 |
| **RV/TLC** | 43.2±9.3 | 41.7±8.0 | 40.3±9.2 | 0.303 |

LCI: lung clearance index; RV/TLC: the ratio of residual volume to total lung capacity; nLCI: normalized lung clearance index

All analyses were explorative in nature because we did not adjust for multiple comparisons.

**Table B. Discriminative performance of different parameters in discriminating moderate-to-severe from mild bronchiectasis and severe from mild-to-moderate bronchiectasis**

|  | **Parameter** | **Cut-off** | **Area under curve** | | **Sensitivity** | **Specificity** | **Correctly classified** |
| --- | --- | --- | --- | --- | --- | --- | --- |
|  |  |  | **Mean** | **95%CI** |  |  |  |
| **Moderate-to-severe vs mild bronchiectasis** | **LCI** | 13.2 | 0.731 | 0.642, 0.821 | 0.808 | 0.611 | 72.4% |
|  | **RV/TLC** | 38.0% | 0.622 | 0.524, 0.720 | 0.726 | 0.519 | 62.2% |
|  | **nLCI** | 31.7 | 0.633 | 0.536, 0.730 | 0.753 | 0.500 | 64.6% |
| **Mild-to-moderate vs severe bronchiectasis** | **LCI** | 13.2 | 0.703 | 0.613, 0.793 | 0.884 | 0.500 | 63.0% |
|  | **RV/TLC** | 42.3% | 0.693 | 0.594, 0.791 | 0.698 | 0.643 | 66.1% |
|  | **nLCI** | 31.6 | 0.560 | 0.454, 0.665 | 0.767 | 0.417 | 53.5% |

LCI: lung clearance index; RV/TLC: the ratio of residual volume to total lung capacity; nLCI: normalized lung clearance index

**Table C. Diagnostic value of LCI, RV/TLC and nLCI to discriminate patients with or without FEV_1_** >80% predicted

| **Parameters** | **Area under curve** | **P value** | **95% confidence interval** | | **Cut-off** | **Sensitivity** | **Specificity** |
| --- | --- | --- | --- | --- | --- | --- | --- |
|  |  |  | **Lower limit** | **Upper limit** |  |  |  |
| **LCI** | 0.81 | <.01 | 0.73 | 0.90 | 13.1 | 0.80 | 0.82 |
| **RV/TLC** | 0.72 | .05 | 0.62 | 0.82 | 39.4 | 0.68 | 0.73 |
| **nLCI** | 0.62 | .05 | 0.51 | 0.72 | 38.0 | 0.45 | 0.79 |

LCI: lung clearance index; RV/TLC: the ratio of residual volume to total lung capacity; nLCI: normalized lung clearance index

**Table D. Comparison and correlation of LCI and RV/TLC** in different subgroups

|  | **No.** | **LCI**  **Median (95%CI)** | **RV/TLC (%)**  **Median (95%CI)** | **r value of correlation **** | **P value of correlation **** |
| --- | --- | --- | --- | --- | --- |
| **All patients** | 127 | 14.6 (12.0, 17.6) | 41.5 (34.8, 47.3) | 0.42 | <0.01 |
| **Mild** | 41 | 12.4 (10.6, 16.1) | 38.0 (33.0, 45.8) | 0.49 | <0.01 |
| **Moderate** | 43 | 14.6 (11.8, 17.6) | 39.4 (33.3, 46.1) | 0.25 | 0.12 |
| **Severe** | 43 | 16.7 (13.8, 18.8) | 45.6 (38.7, 53.0) | 0.45 | <0.01 |
| **P value *** | - | <0.01 | <0.01 | - | - |

95%CI: 95% confidence interval

LCI: lung clearance index; RV/TLC: the ratio of residual volume to total lung capacity

*Comparison among patients with mild bronchiectasis (BSI less than 5), moderate bronchiectasis (BSI of 5 or greater and 8 or lower), and those with severe bronchiectasis (BSI of 9 or greater).

** Correlation between LCI and RV/TLC in different subgroups was analyzed with partial correlation model, adjusting for the patient’s age, sex and body-mass index

**Table E. Correlation between FEV_1_**/FVC% and LCI, RV/TLC or nLCI in different subgroups

| **Correlation category** | **Bronchiectasis severity** | **No.** | **r Value of Correlation *** | **P Value of Correlation *** |
| --- | --- | --- | --- | --- |
| **FEV_1_/FVC% and LCI** | **All patients** | 127 | -0.54 | <0.01 |
|  | **Mild** | 37 | -0.48 | <0.01 |
|  | **Moderate** | 39 | -0.62 | <0.01 |
|  | **Severe** | 39 | -0.44 | <0.01 |
| **FEV_1_/FVC% and RV/TLC** | **All patients** | 127 | -0.42 | <0.01 |
|  | **Mild** | 37 | -0.45 | <0.01 |
|  | **Moderate** | 39 | -0.36 | 0.02 |
|  | **Severe** | 39 | -0.44 | <0.01 |
| **FEV_1_/FVC% and nLCI** | **All patients** | 127 | -0.24 | 0.01 |
|  | **Mild** | 37 | -0.20 | 0.23 |
|  | **Moderate** | 39 | -0.30 | 0.06 |
|  | **Severe** | 39 | -0.08 | 0.64 |

LCI: lung clearance index

* Correlation between FEV_1_/FVC% and LCI, RV/TLC or nLCI in different subgroups was analyzed with partial correlation model, adjusting for the age, sex and body-mass index.

**Table F. Fixed-effect estimates in multivariate linear mixed model of the clinical variable attributes’ impacts on LCI, RV/TLC and nLCI in patients with mild bronchiectasis**

|  | **LCI** | | |  | **RV/TLC** | | |  | **nLCI** | | |
| --- | --- | --- | --- | --- | --- | --- | --- | --- | --- | --- | --- |
|  | **Estimate** | **P value** | **95% CI** |  | **Estimate** | **P value** | **95% CI** |  | **Estimate** | **P value** | **95% CI** |
| **Intercept** | **17.70** | **<0.001** | **14.78, 20.63** |  | **50.00** | **<0.001** | **47.08, 52.93** |  | **36.37** | **<0.001** | **33.44, 39.29** |
| **No. of bronchiectatic lobes** | **-0.80** | **<0.001** | **-1.19, -0.40** |  | **-0.41** | **0.041** | **-0.81, -0.02** |  | **-1.26** | **<0.001** | **-1.66, -0.86** |
| **HRCT total score*** | **0.60** | **<0.001** | **0.38, 0.81** |  | **-0.24** | **0.031** | **-0.46, -0.02** |  | **1.47** | **<0.001** | **1.25, 1.69** |
| **Age** | **0.04** | **0.01** | **0.01, 0.07** |  | **0.29** | **<0.001** | **0.26, 0.32** |  | **-0.18** | **<0.001** | **-0.21, -0.15** |
| **FEV_1_ predicted%** | **-0.12** | **<0.001** | **-0.15, -0.10** |  | **-0.26** | **<0.001** | **-0.28, -0.24** |  | **-0.08** | **<0.001** | **-0.11, -0.06** |
| **Sex** | - | - | - |  | - | - | - |  | - | - | - |
| Males | Reference | Reference | Reference |  | Reference | Reference | Reference |  | Reference | Reference | Reference |
| Females | **1.21** | **<0.001** | **0.51, 1.91** |  | **2.82** | **<0.001** | **2.12, 3.52** |  | **1.43** | **<0.001** | **0.73, 2.13** |
| ***Pseudomonas aeruginosa* colonization** | - | - | - |  | - | - | - |  | - | - | - |
| Yes | Reference | Reference | Reference |  | Reference | Reference | Reference |  | Reference | Reference | Reference |
| No | 0.52 | 0.370 | -0.61, 1.65 |  | -1.12 | 0.052 | -2.26, 0.01 |  | **2.77** | **<0.001** | **1.63, 3.90** |
| **Cystic bronchiectasis** | - | - | - |  | - | - | - |  | - | - | - |
| Yes | Reference | Reference | Reference |  | Reference | Reference | Reference |  | Reference | Reference | Reference |
| No | **2.11** | **<0.001** | **1.28, 2.93** |  | **-1.48** | **<0.001** | **-2.30, -0.66** |  | **5.98** | **<0.001** | **5.16, 6.80** |
| **Mosaicism** | - | - | - |  | - | - | - |  | - | - | - |
| Yes | Reference | Reference | Reference |  | Reference | Reference | Reference |  | Reference | Reference | Reference |
| No | -0.73 | 0.062 | -1.51, 0.04 |  | **-3.64** | **<0.001** | **-4.41, -2.86** |  | **1.79** | **<0.001** | **1.02, 2.56** |

95%CI: 95% confidence interval

LCI: lung clearance index; RV/TLC: the ratio of residual volume to total lung capacity; nLCI: normalized lung clearance index

Data in bold indicated the statistical analyses with significance.

*Modified Reiff score

**Table G. Fixed-effect estimates in multivariate linear mixed model of the clinical variable attributes’ impacts on LCI, RV/TLC and nLCI in patients with moderate bronchiectasis**

|  | **LCI** | | |  | **RV/TLC** | | |  | **nLCI** | | |
| --- | --- | --- | --- | --- | --- | --- | --- | --- | --- | --- | --- |
|  | **Estimate** | **P value** | **95% CI** |  | **Estimate** | **P value** | **95% CI** |  | **Estimate** | **P value** | **95% CI** |
| **Intercept** | **18.27** | **<0.001** | **16.32, 20.22** |  | **27.80** | **<0.001** | **25.85, 29.75** |  | **58.81** | **<0.001** | **56.85, 60.76** |
| **No. of bronchiectatic lobes** | **0.47** | **0.040** | **0.02, 0.92** |  | -0.22 | 0.344 | -0.67, 0.23 |  | **1.32** | **<0.001** | **0.87, 1.77** |
| **HRCT total score*** | 0.11 | 0.337 | -0.11, 0.32 |  | **0.75** | **<0.001** | **0.54, 0.97** |  | **-0.36** | 0.001 | **-0.58, -0.14** |
| **Age** | 0.02 | 0.114 | -0.01, 0.04 |  | **0.28** | **<0.001** | **0.26, 0.30** |  | **-0.21** | **<0.001** | **-0.23, -0.19** |
| **FEV_1_ predicted%** | **-0.07** | **<0.001** | **-0.09, -0.05** |  | **-0.11** | **<0.001** | **-0.13, -0.09** |  | **-0.06** | **<0.001** | **-0.08, -0.04** |
| **Sex** | - | - | - |  | - | - | - |  | - | - | - |
| Males | Reference | Reference | Reference |  | Reference | Reference | Reference |  | Reference | Reference | Reference |
| Females | -0.50 | 0.177 | -1.21, 0.22 |  | **-2.01** | **<0.001** | **-2.73, -1.29** |  | **1.81** | **<0.001** | **1.09, 2.53** |
| ***Pseudomonas aeruginosa* colonization** | - | - | - |  | - | - | - |  | - | - | - |
| Yes | Reference | Reference | Reference |  | Reference | Reference | Reference |  | Reference | Reference | Reference |
| No | **-2.55** | **<0.001** | **-3.44, -1.65** |  | **2.65** | **<0.001** | **1.75, 3.55** |  | **-10.85** | **<0.001** | **-11.74, -9.95** |
| **Cystic bronchiectasis** | - | - | - |  | - | - | - |  | - | - | - |
| Yes | Reference | Reference | Reference |  | Reference | Reference | Reference |  | Reference | Reference | Reference |
| No | **1.61** | **<0.001** | **0.74, 2.49** |  | **-1.63** | **<0.001** | **-2.51, -0.76** |  | **5.82** | **<0.001** | **4.95, 6.70** |
| **Mosaicism** | - | - | - |  | - | - | - |  | - | - | - |
| Yes | Reference | Reference | Reference |  | Reference | Reference | Reference |  | Reference | Reference | Reference |
| No | **-1.84** | **<0.001** | **-2.71, -0.97** |  | **3.08** | **<0.001** | **2.21, 3.95** |  | **-9.24** | **<0.001** | **-10.10, -8.37** |

95%CI: 95% confidence interval

LCI: lung clearance index; RV/TLC: the ratio of residual volume to total lung capacity; nLCI: normalized lung clearance index

Data in bold indicated the statistical analyses with significance.

*Modified Reiff score

**Table H. Fixed-effect estimates in multivariate linear mixed model of the clinical variable attributes’ impacts on LCI, RV/TLC and nLCI in patients with severe bronchiectasis**

|  | **LCI** | | |  | **RV/TLC** | | |  | **nLCI** | | |
| --- | --- | --- | --- | --- | --- | --- | --- | --- | --- | --- | --- |
|  | **Estimate** | **P value** | **95% CI** |  | **Estimate** | **P value** | **95% CI** |  | **Estimate** | **P value** | **95% CI** |
| **Intercept** | **14.11** | **<0.001** | **11.56, 16.65** |  | **33.98** | **<0.001** | **31.44, 36.52** |  | **46.83** | **<0.001** | **44.29, 49.37** |
| **No. of bronchiectatic lobes** | 0.21 | 0.311 | -0.19, 0.60 |  | **0.80** | **<0.001** | **0.40, 1.20** |  | **-0.85** | **<0.001** | **-1.24, -0.45** |
| **HRCT total score*** | **0.40** | **<0.001** | **0.26, 0.55** |  | **0.33** | **<0.001** | **0.18, 0.47** |  | **0.67** | **<0.001** | **0.52, 0.82** |
| **Age** | **0.09** | **<0.001** | **0.06, 0.11** |  | **0.31** | **<0.001** | **0.29, 0.33** |  | **-0.15** | **<0.001** | **-0.17, -0.13** |
| **FEV_1_ predicted%** | **-0.11** | **<0.001** | **-0.13, -0.09** |  | **-0.21** | **<0.001** | **-0.23, -0.19** |  | **-0.05** | **<0.001** | **-0.07, -0.03** |
| **Sex** | - | - | - |  | - | - | - |  | - | - | - |
| Males | Reference | Reference | Reference |  | Reference | Reference | Reference |  | Reference | Reference | Reference |
| Females | 0.18 | 0.596 | -0.48, 0.83 |  | **3.30** | **<0.001** | **2.64, 3.95** |  | **-4.13** | **<0.001** | **-4.78, -3.48** |
| ***Pseudomonas aeruginosa* colonization** | - | - | - |  | - | - | - |  | - | - | - |
| Yes | Reference | Reference | Reference |  | Reference | Reference | Reference |  | Reference | Reference | Reference |
| No | **-0.93** | **0.003** | **-1.54, -0.31** |  | **-1.14** | **<0.001** | **-1.76, -0.53** |  | **-0.94** | **0.003** | **0.32, 1.55** |
| **Cystic bronchiectasis** | - | - | - |  | - | - | - |  | - | - | - |
| Yes | Reference | Reference | Reference |  | Reference | Reference | Reference |  | Reference | Reference | Reference |
| No | **-1.29** | **0.009** | **-2.26, -0.32** |  | **-4.37** | **<0.001** | **-5.34, -3.40** |  | **1.10** | **0.025** | **0.14, 2.07** |
| **Mosaicism** | - | - | - |  | - | - | - |  | - | - | - |
| Yes | Reference | Reference | Reference |  | Reference | Reference | Reference |  | Reference | Reference | Reference |
| No | **1.42** | **0.007** | **0.38, 2.46** |  | -0.65 | 0.221 | -1.68, 0.39 |  | **2.55** | **<0.001** | **1.51, 3.58** |

95%CI: 95% confidence interval

LCI: lung clearance index; RV/TLC: the ratio of residual volume to total lung capacity; nLCI: normalized lung clearance index

Data in bold indicated the statistical analyses with significance.

*Modified Reiff score
