# Supplementary figures and images for: Residual volume/total lung capacity ratio confers limited additive significance to lung clearance index for assessment of adults with bronchiectasis
Source: PLoS One. 2017 Sep 8;12(9):e0183779. doi: 10.1371/journal.pone.0183779 (PMC5590849; doi:10.1371/journal.pone.0183779)

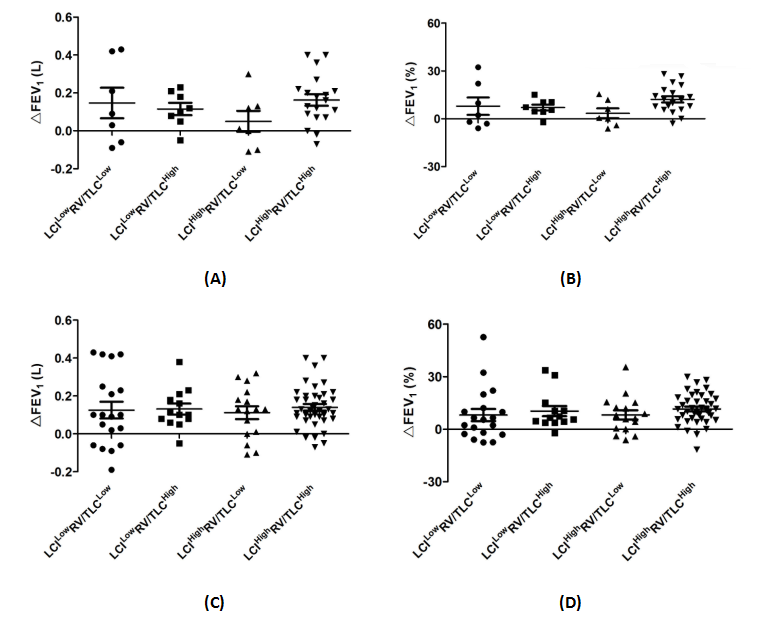

Supplement: S2 Fig — (A). Difference between pre- and post-bronchodilator FEV1 (expressed in absolute values) in bronchiectasis patients who underwent bronchodilation test within the same day of multiple-breath nitrogen washout test and spirometry; Statistical analyses were done in 7, 8, 7 and 19 patients with available data (bronchodilator tests performed within the same day of the multiple-breath washout test) in groups LCILowRV/TLCLow, LCILowRV/TLCHigh, LCIHighRV/TLCLow, and LCIHighRV/TLCHigh, respectively. Overall, no significant difference was observed when comparing the data among the four subgroups. (B). Difference between pre- and post-bronchodilator FEV1 (expressed in percentage) in bronchiectasis patients who underwent bronchodilation test within the same day of multiple-breath nitrogen washout test and spirometry; Statistical analyses were done in 7, 8, 7 and 19 patients with available data (bronchodilator tests performed within the same day of the multiple-breath washout test) in groups LCILowRV/TLCLow, LCILowRV/TLCHigh, LCIHighRV/TLCLow, and LCIHighRV/TLCHigh, respectively. Overall, no significant difference was observed when comparing the data among the four subgroups. (C). Difference between pre- and post-bronchodilator FEV1 (expressed in absolute values) in all bronchiectasis patients who had ever undergone bronchodilation test (either at the baseline visit or within the previous 2 years); Statistical analyses were done in 19, 13, 16 and 40 patients with available data (bronchodilator tests performed within the same day of the multiple-breath washout test) in groups LCILowRV/TLCLow, LCILowRV/TLCHigh, LCIHighRV/TLCLow, and LCIHighRV/TLCHigh, respectively. Overall, no significant difference was observed when comparing the data among the four subgroups. (D). Difference between pre- and post-bronchodilator FEV1 (expressed in percentage) in all bronchiectasis patients who had ever undergone bronchodilation test (either at the baseline visit or within the previous 2 years); St [file pone.0183779.s005.tif]
